# Neuroendocrine tumor of the pancreas with rhabdoid feature

**DOI:** 10.1007/s00428-018-2398-x

**Published:** 2018-06-24

**Authors:** Tetsuyuki Miyazaki, Shinichi Aishima, Minoru Fujino, Keigo Ozono, Yuichiro Kubo, Yasuhiro Ushijima, Takashi Osoegawa, Eikichi Ihara, Itou Tetsuhide, Takao Ohtsuka, Masafumi Nakamura, Yoshinao Oda

**Affiliations:** 1 Department of Anatomic Pathology, Graduate School of Medical Sciences, Kyushu University, 3-1-1 Maidashi, Fukuoka, 812-8582 Japan; 2 Department of Surgery and Oncology, Graduate School of Medical Sciences, Kyushu University, Fukuoka, Japan; 3 Department of Pathology and Microbiology, Faculty of Medicine, Saga University, Saga, Japan; 4 Department of Clinical Radiology, Graduate School of Medical Sciences, Kyushu University, Fukuoka, Japan; 5 Department of Medicine and Bioregulatory Science, Graduate School of Medical Sciences, Kyushu University, Fukuoka, Japan

**Keywords:** Neuroendocrine tumor, Rhabdoid feature, Cytoplasmic inclusion, Pancreas

## Abstract

Imaging of a 53-year-old Japanese man revealed two tumors in the liver and a tumor in the head of the pancreas with a swelling lymph node. A needle biopsy for the liver tumors was performed, revealing a neuroendocrine tumor. Enucleation, lymphadenectomy, and partial hepatectomy were performed. The microscopic examination identified many tumor cells with intracytoplasmic inclusions arranged in a nested, cord, or tubular fashion. The intracytoplasmic inclusions displayed densely eosinophilic globules and displaced the nuclei toward the periphery, which constitutes “rhabdoid” features. The tumor cells were positive for synaptophysin and weakly positive for NCAM, but negative for chromogranin A. Epithelial markers (AE1/AE3 and CAM5.2) accentuated intracytoplasmic globules. Pancreatic neuroendocrine tumors with rhabdoid features are very rare. Generally, rhabdoid features are aggressive and dedifferentiated characteristics of various types of tumor. Pancreatic neuroendocrine tumors containing rhabdoid cells tend to display extrapancreatic spread at the time of presentation, although some of these tumors with rhabdoid features are not always associated with aggressive behavior.

## Introduction

Neuroendocrine tumors of the pancreas are rare neoplasms that account for 1–2% of all pancreatic tumors [1]. In the majority of cases, the tumors are characterized by their distinctive “neuroendocrine” appearance, reminiscent of neuroendocrine neoplasms in other organs. Neuroendocrine tumors in the pancreas may show variable morphological alterations, including oncocytic change [2, 3], clear cytoplasm [4, 5], spindle cell morphology [6], and rhabdoid features [7, 8]. Neuroendocrine tumors showing rhabdoid features have been defined based on the cells with deeply eosinophilic cytoplasmic inclusions and a peripherally displaced nucleus that contains a prominent nucleolus [9–11].

We report the case of a patient’s primary neuroendocrine tumor of the pancreas with rhabdoid features, accompanied by multiple liver metastases. We also review the relevant literature.

## Case report

**Fig. 1:**
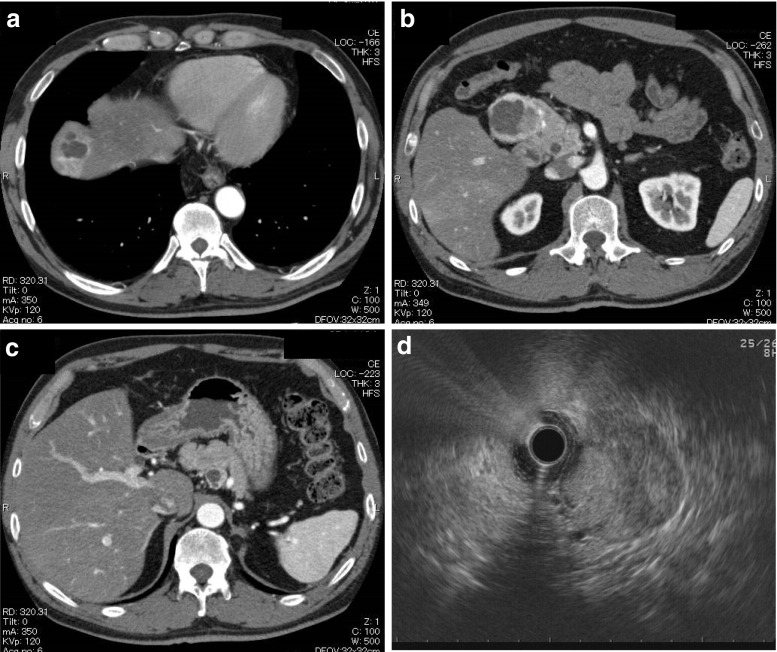
Imaging studies. **a**–**c** Dynamic contrast-enhanced CT scan during the artery phase showed a well-enhanced mass with cystic component in an anterosuperior segment (S8) of the liver (**a**), the head of the pancreas (**b**), and the posterior portion of the body of the pancreas (**c**). **d** Endoscopic ultrasound (EUS) showed a 4.1-cm hypoechoic and well-demarcated mass in the parenchyma of the pancreatic head

A 53-year-old Japanese man with a liver tumor was referred to our hospital for further investigation. A hypervascular mass had been detected in the left lobule of the liver by computer tomography (CT) for other disease. He had a history of hypertension, but no history of hereditary disease such as Von Hippel-Lindau disease nor family history of the patients. The results of a physical examination were unremarkable, and no notable symptoms were present. The biochemical tests revealed slightly elevated levels of alanine aminotransferase (49 U/L) and lactate dehydrogenase (250 U/L). The serum gastrin level (312 pg/ml) was increased. An abdominal contrast-enhanced CT examination confirmed a 4-cm vascular and cystic mass in the anterosuperior segment of the right hepatic lobe and a 0.5-cm vascular lesion in antero/posterosuperior segment of the right hepatic lobe (Fig. 1a). In addition, a 4-cm vascular and cystic mass in the head of the pancreas and a 2-cm vascular and cystic mass in the posterior portion of the body of the pancreas were found (Fig. 1b, c).

Endoscopic ultrasonography (EUS) showed a 4.1-cm well-demarcated hypoechoic mass in the pancreatic head (Fig. 1d) and a 1.9-cm well-demarcated hypoechoic mass in the pancreatic body. High blood flow was detected in the mass by color Doppler ultrasonography. At the previous hospital, a needle biopsy for liver tumors was performed, and it showed that atypical cells with hyperchromatic nuclei and eosinophilic cytoplasm were arranged in a nested fashion. Immunohistochemically, atypical cells were positive for CAM5.2, synaptophysin, chromogranin A, and glucagon and negative for CK7, CK20, AFP, vimentin, CD34, desmin, c-kit, insulin, gastrin, and somatostatin. These results were suggestive of a metastatic neuroendocrine tumor. We performed enucleation for the pancreatic tumor together with lymph node dissection and partial hepatectomy. The postoperative course was uneventful, and the patient was discharged 36 days after the operation. The patient is alive 4.5 years after surgery without evidence of recurrence or metastasis.

## Immunohistochemical procedures

For the histological examination, the tissue samples were fixed in 10% formalin and embedded in paraffin, cut into 3-μm-thick sections, and stained with hematoxylin and eosin. A periodic acid Schiff preparation, with and without diastase digestion, and Southgate’s mucicarmine staining were also done. For the immunohistochemical staining, 3-μm-thick tissue sections were deparaffinized in xylene and dehydrated in ethanol. Endogenous peroxidase activity was blocked by incubation in methanol containing 0.3% H_2_O_2_ for 30 min. Antigen retrieval was achieved by microwave heating in 10 mM citrate buffer (pH 6.0 or 9.0), or Target Retrieval Solution (DakoCytomation, Glostrup, Denmark). The slides were incubated with primary antibodies for 90 min at room temperature and then incubated with biotin-free horseradish peroxidase enzyme-labeled polymer (Envision+ System, DakoCytomation) for 40 min at room temperature. The labeled antigens were visualized by 3,3′-diaminobenzidine tetrahydrochloride as a chromogen. Counterstaining was done with hematoxylin. All of the antibodies were used with the appropriate positive control.

The primary antibodies used for the immunohistochemical analysis were as follows: synaptophysin (1:50; Dako, Glostrup, Denmark), chromogranin A (1:1500; Dako), AE1/AE3 (1:400; Dako), CAM5.2 (1:20; Becton Dickinson, Lincoln Park, NJ), cytokeratin 7 (1:50; Dako), cytokeratin 19 (1:100; Boehringer Mannheim, Gaithersburg, MD), EMA (1:400; Dako), trypsin (1:1000; Chemicon, Billerica, MA), β-catenin (1:200; Transduction Laboratories, Lexington, KY), NCAM (1:50; Novocastra, Newcastle, UK), insulin (1:1; Nichirei Bioscience, Tokyo), glucagon (1:1; Nichirei Bioscience), gastrin (1:400; Dako), somatostatin (1:100; Biomol, Hamburg, Germany), Ki-67 (1:100; Dako), vimentin (1:25; Dako), hepatocyte (1:200; DakoCytomation), BAF47/INI1 (1:50; BD Bioscience, Franklin Lakes, NJ), BAF155 (1:50; Santa Cruz Biotechnology, Santa Cruz, CA), BAF170 (1:100; Santa Cruz Biotechnology), ARID1A (1:500; Sigma, St. Louis, MO), BRG-1 (1:25; Santa Cruz Biotechnology), and p53 (1:500; Calbiochem, Darmstadt, Germany). For the p53 immunolabeling, focal acinar and ductal cells with nuclear p53 immunolabeling served as an internal control. The p53 immunolabeling was interpreted as “high expression” when the neoplastic cells showed robust nuclear accumulation in ≥ 30% of the neoplastic cells. Immunohistochemical labeling of high p53 expression corresponded to mutational inactivation [12]. The MIB-1 labeling index (MIB-1 LI) was determined by counting the positively stained nuclei in at least 500 tumor cells.

## Gross and histologic features

**Fig. 2:**
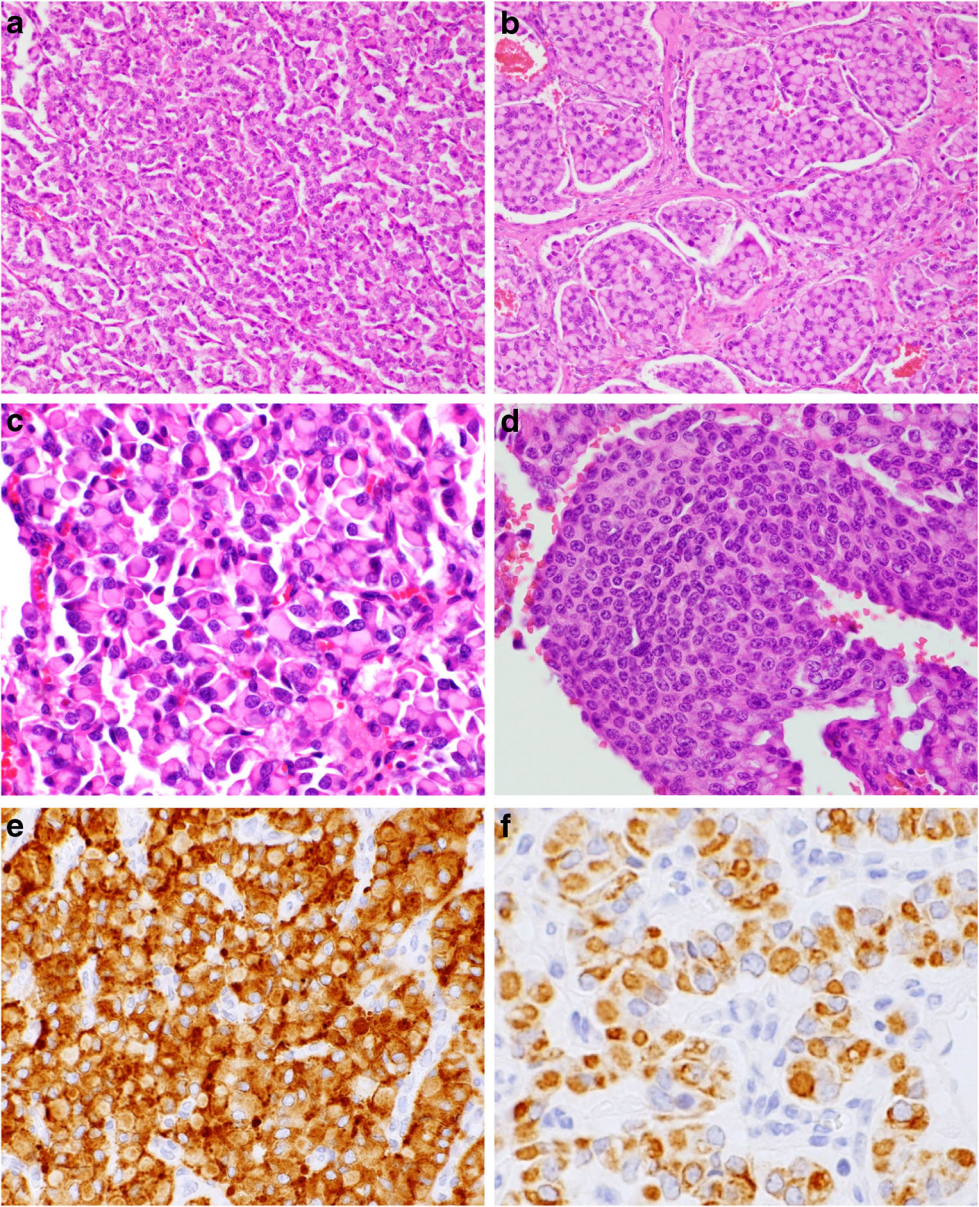
Histological findings for the tumor located in the pancreas head (**a**, **c**–**f**) and liver (**b**). **a** Tumor cells arranged in a cord fashion. **b** Tumor cells arranged in a nested fashion. **c** Tumor cells with abundant cytoplasm and densely eosinophilic globular inclusions that displaced the nuclei toward the periphery. **d** Uniform cells arranged in a nested fashion, demonstrating typical features of a pancreatic neuroendocrine tumor. **e** Tumor cells were positive for synaptophysin. **f** Immunohistochemically, intracytoplasmic globular inclusions were positive for AE1/AE3. Original magnification × 200 (**a**, **b**), × 400 (**c**–**f**)

Resected specimens showed solid, partly cystic and brown/tan to white color with hemorrhage. The size of the pancreatic tumor was 57 × 40 mm, and the sizes of the three liver tumors were 50 × 20 mm, 10 × 6 mm, and 5 × 4 mm. Cystic component was also found in a lymph node and the liver. In both the pancreas and liver, the tumor cells were arranged in a nested, cord, or tubular fashion (Fig. 2a, b). Tumor cells had abundant cytoplasm and lightly or densely eosinophilic globular inclusions that displaced the nuclei toward the periphery (Fig. 2c). The nuclei were round to oval with dispersed (“salt and pepper”) chromatin and nuclear pleomorphism (Fig. 2c). Tumor cells showed mitoses at the rate of one per 10 high-power fields (HPF) in the pancreatic tumor, while six per 10 HPF in the liver tumor. There was an area of moderate necrosis in the pancreatic tumor, but not in the liver tumor. Vessel invasion of tumor cells was observed in the pancreatic tumor and the liver. In part, there were also typical features of a pancreatic neuroendocrine tumor, characterized by a nesting arrangement of uniform cells (Fig. 2d), but tumor cells with intracytoplasmic globules accounted for 80% of the total tumor cell population in the pancreas and 90% of the total tumor cell population in the liver. The rhabdoid features between primary and metastatic tumors were not changed. Hyaline inclusions were negative with the periodic acid Schiff, with and without diastase digestion, and mucin stains.

Immunohistochemically, pancreatic tumor cells were positive for epithelial markers (AE1/AE3 and CAM5.2), CK19, EMA, synaptophysin (Fig. 2e), somatostatin, INI1, BAF155, BAF170, and ARID1A; weakly positive for NCAM, gastrin, and BRG-1; and negative for chromogranin A, β-catenin, trypsin, glucagon, insulin, and vimentin. The p53 immunolabeling for the pancreatic tumor tissue was low expression. AE1/AE3 (Fig. 2f) and CAM5.2 highlighted intracytoplasmic globules. In addition, tumor cells in the liver were positive for chromogranin A and focally positive for HepPar-1. The MIB-1 labeling index was approx. 3% in the pancreas tumor and 2% in the liver tumor. We diagnosed the tumor as a primary pancreatic neuroendocrine tumor, G2, accompanied by metastatic liver tumors and lymph nodes.

## Discussion

The term “rhabdoid” has been used to describe tumors composed of cells with intensely eosinophilic/acidophilic globular cytoplasmic inclusions which are similar to rhabdomyoblasts [9–11]. Electron microscopy studies have shown that the rhabdoid inclusions of pancreatic endocrine tumors are composed of whorls of intermediate filaments that entrapped variable numbers of neurosecretory granules and that in some inclusions, the intermediate filaments and neurosecretory granules are accompanied by rough and smooth endoplasmic reticulum profiles [8]. The whorls of intermediate filaments are mainly keratin filaments, as demonstrated by the positive immunohistochemical staining for keratin and not vimentin [8].

In follicular thyroid carcinomas, the rhabdoid inclusions are vimentin-positive but lack immunoreactivity for cytokeratins, thyroglobulin, smooth-muscle actin, and desmin [13]. By electron microscopy, the eosinophilic inclusions were revealed to be composed of whorls of intermediate filaments that displaced cytoplasmic organelles and the nuclei to the periphery [13].

To the best of our knowledge, there have been only four previous reports regarding such a characteristic morphology of inclusions in a pancreatic neuroendocrine tumor. One report indicated that large intracytoplasmic inclusions occur in approx. 16% of pancreatic neuroendocrine tumors [14]. We conducted a search of the records of the Department of Surgery and Oncology, Kyushu University (1991–2010) for cases of pancreatic neuroendocrine tumor or carcinoma, and we retrieved a total of 72 cases. We reviewed the hematoxylin-eosin-stained slides of all cases, but no case of neuroendocrine tumor with rhabdoid features was found.

It has been proposed that rhabdoid morphology is associated with an aggressive biologic behavior based on lymph node metastasis, local invasion, and early recurrence [10, 13, 15–17]; however, we were unable to find any report regarding other neuroendocrine tumors other than those in the lung and pancreas. We identified only two cases of a lung neuroendocrine tumor with rhabdoid features. One patient had local invasion and died due to the tumor within 1.5 months after the operation [16]. The other patient died because of the tumor 3 months post-surgery [17]. In other neuroendocrine tumors except for those in the lung and pancreas, the biologic behavior of neuroendocrine tumors with rhabdoid features may be unclear.

The clinical features of the cases in the four previous reports and the present case are summarized in Table 1. The patients were six men and six women 37–79 years of age. Eight of the 12 cases (66.7%) showed evidence of metastatic spread (either peripancreatic fat, lymph nodes, or liver), although the tumors in the metastatic cases were classified as a neuroendocrine tumor, G1 or G2, according to the current WHO classification [1]. Six of the 12 cases (50%) were alive and free of disease 2–5 years after surgery. Though the follow-up periods were not available in three cases, for the two patients who died due to tumor, the overall survival periods were 24 and 73 months after diagnosis (Table 1). Not many of the pancreatic neuroendocrine tumor patients containing rhabdoid cells died due to the tumor early after the operation. Pancreatic endocrine tumors containing rhabdoid cells seem to display extrapancreatic spread at the time of presentation while lacking the histomorphological features associated with aggressive tumors (large size, necrosis, high mitotic count, and high proliferate rate) [14]. In our case, the comparatively low Ki-67 index for the proliferation marker may be consistent with an indolent nature of the lesion.

**Table 1:** The clinicopathologic features of reported cases of neuroendocrine tumor of the pancreas with rhabdoid features

Case	Age	Sex	Location	Size (cm)	Gross appearance	Metastases	Mitosis (HPF)	Ki-67 labeling index (%)	MEN-1	Treatment	Additional treatment	Outcome	Reference
1	37	M	Tail	3.4	Poorly circumscribed, infiltrative mass	Periaortic lymph nodes	Very scant	ND	NA	Partial pancreatectomy + lymphadenectomy	No	Alive, no recurrence, 2 years after surgery	[7]
2	55	F	Head	2.5	Well-circumscribed mass	Multiple liver metastases	NA	ND	NA	Pancreaticoduodenectomy + partial hepatectomy	–	Dead, peritonitis 1 month after surgery	[7]
3	58	M	Head	4	Well-circumscribed mass	No	Rare	ND	NA	Pancreaticoduodenectomy	No	Alive, no recurrence, 4 years after surgery	[7]
4	79	F	Tail	4.5	Well-circumscribed mass	No	< 2/10	ND	NA	Distal pancreatectomy + splenectomy	No	Alive, no recurrence, 5 years after surgery	[7]
5	60	M	Head	NA	NA	Bilobar liver metastases	ND	< 1%	NA	Radiation + chemotherapy + alcohol injection	NA	Dead, 73 months after the initial diagnosis	[8]
6	35	M	NA	3.2	NA	3 lymph nodes, invasion of peripancreatic soft tissue	≤ 4/10	< 5%	Yes	NA	NA	Alive, no recurrence, after 2 years	[14]
7	55	M	NA	11	NA	9 lymph nodes, invasion of peripancreatic soft tissue	≤ 4/10	< 5%	No	NA	NA	Alive, no recurrence, after 5 years	[14]
8	45	F	NA	5	NA	No	≤ 4/10	< 5%	Yes	NA	NA	No follow-up available	[14]
9	41	F	NA	8	NA	1 lymph node	≤ 4/10	< 5%	No	NA	NA	No follow-up available	[14]
10	67	F	NA	6	NA	1 lymph node	≤ 4/10	< 5%	No	NA	NA	Dead, 2 years after diagnosis	[14]
11	50	F	Head	4.4	Well-circumscribed mass	No	4–9/10	6%	NA	NA	NA	NA	[13]
12	53	M	Head	5.7	Well-circumscribed mass	Multiple liver metastases 4 lymph nodes	1/10	3%	No	Enucleation + lymphadenectomy + partial hepatectomy	No	Alive, no recurrence, 4.5 years after surgery	Present case

*NA* not available, *ND* not detected

For both biopsy and resected specimens in the liver, the tumor cells were positive for chromogranin A, but pancreatic tumor cells were negative for chromogranin A. There were discrepant results of chromogranin A between resected specimens in the pancreas and liver. The reason for discrepancy is that there may be possibility of genetic heterogeneity in the primary pancreatic tumor as pancreatic cancer [18].

The term “rhabdoid,” meaning rhabdomyoblast-like, implies a resemblance to rhabdomyoblasts that is characteristic of aggressive, dedifferentiated malignancies. Pancreatic neuroendocrine tumors containing rhabdoid cells seem to display extrapancreatic spread at the time of presentation, but they are not always associated with aggressive behavior compared to other neoplasms with rhabdoid features.
